# The Heme Connection: Linking Erythrocytes and Macrophage Biology

**DOI:** 10.3389/fimmu.2017.00033

**Published:** 2017-01-24

**Authors:** Md Zahidul Alam, Samir Devalaraja, Malay Haldar

**Affiliations:** ^1^Department of Pathology and Laboratory Medicine, Perelman School of Medicine at the University of Pennsylvania, Philadelphia, PA, USA

**Keywords:** heme, iron-recycling macrophage, erythrophagocytosis, SpiC, Bach1

## Abstract

Erythroid function and development is intimately linked to macrophages. The primary function of erythrocytes is oxygen delivery, which is mediated by iron-containing hemoglobin. The major source of this iron is a recycling pathway where macrophages scavenge old and damaged erythrocytes to release iron contained within the heme moiety. Macrophages also promote erythropoiesis by providing a supportive niche in the bone marrow as an integral component of “erythorblastic islands.” Importantly, inflammation leads to alterations in iron handling by macrophages with significant impact on iron homeostasis and erythropoiesis. The importance of macrophages in erythropoiesis and iron homeostasis is well established and has been extensively reviewed. However, this developmental relationship is not one way, and erythrocytes can also regulate macrophage development and function. Erythrocyte-derived heme can induce the development of iron-recycling macrophages from monocytes, engage pattern recognition receptors to activate macrophages, and act as ligand for specific nuclear receptors to modulate macrophage function. Here, we discuss the role of heme as a signaling molecule impacting macrophage homeostasis. We will review these actions of heme within the framework of our current understanding of the role of micro-environmental factors in macrophage development and function.

## Macrophage Diversity

Macrophages are prominent cells of the innate immune system characterized by their high phagocytic capacity and the ability to process antigens. While best known for their roles in controlling immune responses, they are functionally very versatile with roles in wound repair, tissue morphogenesis, and tissue homeostasis (1, 2). Almost every tissue in the body harbors resident macrophage population that performs tissue-specific homeostatic function (2). Examples include surfactant recycling by alveolar macrophages in the lung and iron recycling by splenic red pulp macrophages (RPM). The functional distinction between tissue-resident macrophages is reflected in their distinct gene-expression profile and dependence on specific transcription factors. As examples, RPM development is dependent on the transcription factor SpiC, Kuffer cells on transcription factor Id3, peritoneal macrophages on transcription factor Gata6, alveolar macrophages on transcription factor PPAR-γ, and splenic marginal zone macrophages on transcription factor Lxrα (3–7). An important question is how divergent tissue-resident macrophages are generated from a common precursor. Recent work suggests a prominent role of the tissue microenvironment in this process. For instance, high levels of heme in the red pulp area of spleen were found to induce the expression of the transcription factor SpiC, which promoted the development of monocytes into RPM (8). Likewise, local retinoic acid levels were found to control the development and localization of peritoneal macrophages (6). An additional line of evidence comes from the finding that tissue macrophages can be reprogramed when transplanted into a new tissue microenvironment (9). Therefore, local microenvironment-associated factors play key role in generating phenotypic and functional diversity of tissue-resident macrophages.

Monocytes were considered to be the primary source of tissue-resident macrophages. However, definitive studies in recent years have changed this paradigm, and we now recognize two distinct precursors of tissue macrophages: circulating monocytes and embryonic precursors that seed various tissue before birth and are maintained by local proliferation (10). Embryonic precursors of tissue macrophages develop independent of hematopoietic stem cells, either from the yolk sac during early stages of embryogenesis or from fetal monocyte-like cells in later stages. In adults, circulating monocytes can differentiate into tissue macrophages, especially during inflammation or tissue injury. Monocytes have a limited lifespan of a few days in the circulation and are continuously replenished from hematopoietic stem cells in the bone marrow (11, 12). The implication of the dual origin (embryonic vs. adult monocyte) on macrophage function is currently not clear. Recent studies suggest that the relative distribution of embryonic and monocyte-derived macrophages in tissue undergoes significant changes with age and pathological conditions. In both instances, the contribution of monocyte-derived macrophages appears to increase (13). Tissue inflammation lead to recruitment of circulating monocytes that differentiate locally into macrophages or closely related antigen-presenting cells known as dendritic cells (DCs) depending on local micro-environmental factors that are not well understood (14).

Macrophage function can also be categorized into pro-inflammatory as embodied by the “M1-polarized” macrophage and anti-inflammatory as represented by the “M2-polarized” macrophage (15). These polarization states represent the two extremes of a functional spectrum from an anti-inflammatory phenotype that support tissue repair and homeostasis to an inflammatory phenotype that induces and support immune responses (16, 17). Tissue macrophages generally have features of M2, while M1 macrophages are generated in inflammatory and infectious settings. Functional polarization of macrophages is driven by various activation signals including cytokines, which has been extensively reviewed elsewhere (15). Notably, the activation state of a macrophage can impact how it will respond to tissue-associated factors. As an example, high levels of heme induce the iron exporter ferroportin (SLC40A1) in macrophages to help recycle iron (18, 19). However, macrophages can strongly downregulate ferroportin if they sense the presence of bacterial pathogens in the environment in an attempt to sequester iron from extracellular pathogens (18, 20, 21). Therefore, macrophages integrate diverse cues from its environment to formulate a context-appropriate response.

In summary, local microenvironment-associated factors have significant impact on macrophage differentiation and function. Additionally, the activation state of the macrophage influences how it will respond to such microenvironment-associated factors.

## Iron-Recycling Macrophages

**Figure 1:**
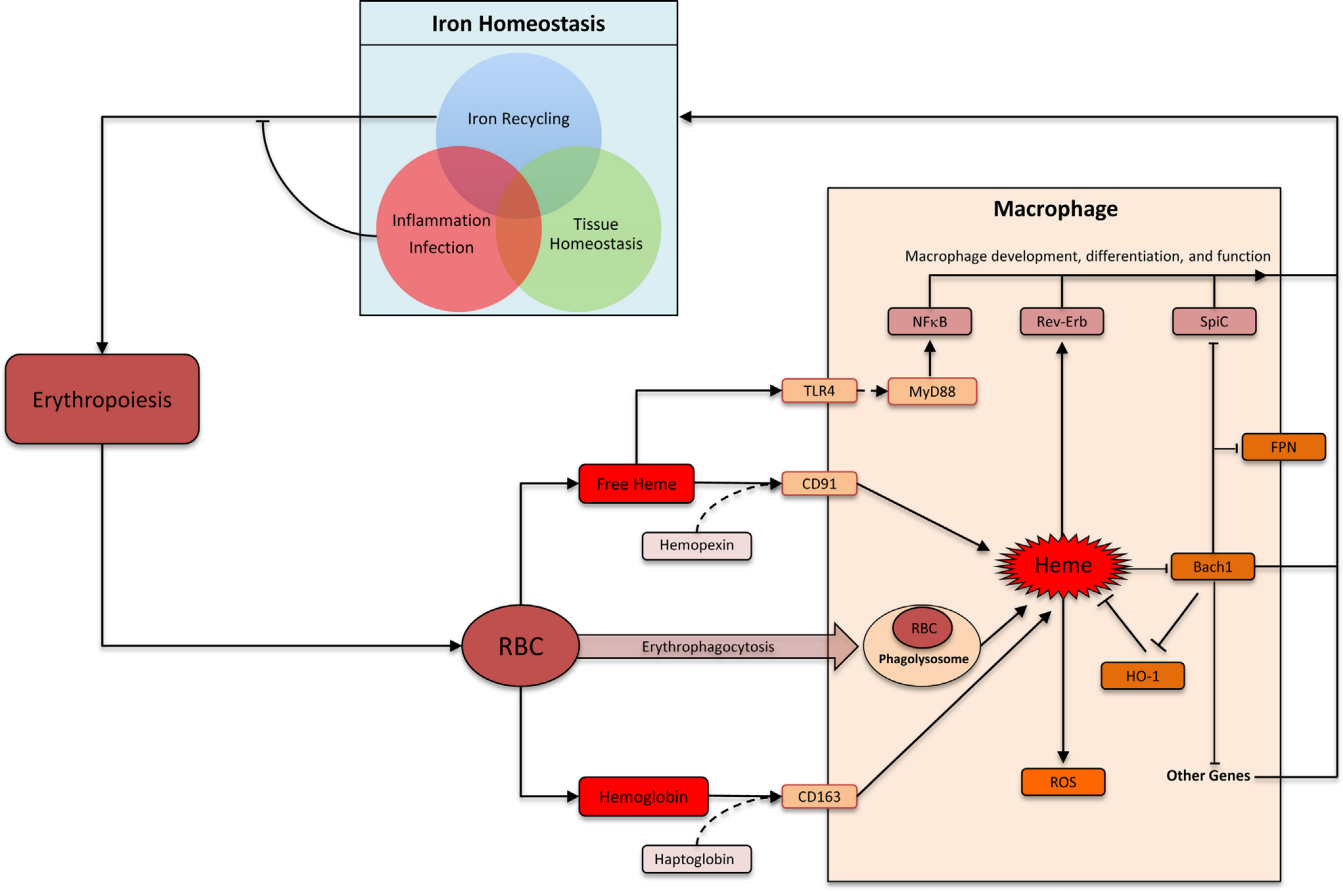
**Heme: linking erythropoiesis and macrophage function**. Abbreviations: RBC, red blood cells; ROS, reactive oxygen species; FPN, ferroportin (Slc40A1); HO-1, heme oxygenase-1. Heme is taken up by macrophages by several distinct pathways. Heme-induced HO-1 leads to heme degradation. Inside macrophages, heme triggers multiple distinct pathways that impacts macrophage function and differentiation. In the context of iron homeostasis, heme-induced functional changes can be divided into three groups: iron recycling at the steady state, iron-sequestration during infection and inflammation, and preventing heme toxicity to maintain tissue homeostasis.

Heme comprises of a protoporphyrin IX ring with an iron atom in the center (22). The capacity of the iron to undergo reversible change in oxidation status is central to heme’s ability to catalyze diverse reactions such as delivery of oxygen to tissue, electron transfer, and oxidation reactions (23). The specific function of heme depends on the protein to which it is attached as a prosthetic group. There are many distinct types of hemoproteins performing various functions. However, the vast majority of the heme in vertebrates is sequestered within hemoglobin and myoglobin in erythrocytes and muscle cells, respectively, which can be released upon damage to these cells (24). Macrophages are the primary cells responsible for the uptake and disposal of heme, which is important for three main reasons: (1) preventing heme and iron-mediated cellular toxicity, (2) recycling iron to sustain erythropoiesis, and (3) preventing pathogen’s access to iron during infection (Figure 1) (25, 26). Each erythrocyte contains approximately 1.2 × 10^9^ heme moieties associated with hemoglobin, and approximately 200 billion erythrocytes reach senescence each day (26). Hemoglobin released from senescent or damaged erythrocytes can be readily oxidized releasing the prosthetic heme group. Free heme can catalyze oxidation of proteins, generate lipid peroxides, and damage DNA through oxidative stress (27). Iron released from heme can also generate harmful free radicals *via* Fenton chemistry (28). Therefore, without a proper disposal mechanism there is a considerable threat from the large amount of heme that can be released from erythrocytes. Alterations in membranes of senescent and damaged erythrocytes are detected by specialized erythrophagocytic macrophages in the spleen, liver, and bone marrow, which remove these erythrocytes before they undergo hemolysis (Figure 1) (29–31). Cell-free hemoglobin and heme binds to carrier proteins haptoglobin and hemopexin, respectively (31). Hemoglobin–haptoglobin and heme–hemopexin complexes are taken up by macrophages *via* cognate receptors CD163 and CD91, respectively (Figure 1) (32, 33). Therefore, macrophages prevent heme-mediated toxicity by phagocytosing old erythrocytes before they rupture as well as by taking up heme and hemoglobin that is already released from erythrocytes.

Heme is metabolized inside macrophages by the sequential activity of Heme oxygenase (HO) and biliverdin reductase (BVR). HO has two isoforms: the inducible HO-1 and the constitutively expressed HO-2 (34). HO-1 levels increase upon heme accumulation in macrophage and catalyze the breakdown of heme into biliverdin. BVR then converts biliverdin to bilirubin, which is exported out of the cells to be incorporated into bile acid in the liver (26, 31, 34). Iron is released upon degradation of heme to bilirubin. This iron can be either stored inside the macrophages in combination with ferritin or exported out of the cells *via* the iron exporter ferroportin (26, 31). The decision to store or release iron depends upon various factors such as iron requirements of the host and the presence or absence of inflammation and infection. At the steady state, the vast majority (>90%) of the body’s iron requirement is provided by this macrophage-based recycling machinery (Figure 1). Inflammation and infection with extracellular pathogens leads to a much greater propensity to store, rather than release, iron inside macrophages (35). One underlying mechanism is the release of hepcidin from hepatocytes in response to inflammation, which leads to internalization and degradation of ferroportin (36). Indeed, such infectious settings promote iron accumulation by macrophages leading to a systemic reduction of circulating iron. This is a form of evolutionarily conserved immune response aimed at limiting pathogen’s access to critical nutrients (35). Therefore, iron recycling by macrophages is carefully regulated to strike a balance between iron requirements of the host, avoiding deleterious effects of heme/iron, and preventing pathogen access to iron.

## Heme-Mediated Regulation of Iron-Recycling Macrophages

Iron recycling by macrophages is a highly specialized function, which involves several closely coordinated steps from heme acquisition and heme degradation to iron storage and release. As the first element in this metabolic cascade, heme regulates many of the subsequent steps in this pathway. Heme can physically bind the transcriptional factor Bach1 (BTB and CNC homology 1) *via* a dipeptide motif of cysteine and proline, which inhibits its functions (37). Bach1 and the closely related Bach2 belong to the basic leucine zipper family of transcription factors, which control gene expression by forming heterodimers with small Maf proteins (38). Bach–Maf heterodimers bind to Maf recognition elements (MARE) in the genome to suppress target genes such as HO1, ferroportin, and ferritin (38). Direct binding of heme to Bach1 inhibits Bach1 activity by (1) altering its DNA binding, (2) promoting its nuclear export, and (3) inducing polyubiquitination and degradation of Bach1 protein (39–42). In the absence of Bach1, Maf binds to transcription factor Nrf2. Nrf2–Maf heterodimers also bind MARE sequences, but leads to target gene activation (38, 43). Therefore, heme levels inside a cell can control the relative abundance of “activating” Nrf2-Maf and “repressive” Bach1–Maf heterodimers. This is a key mechanism by which high levels of environmental heme induce the expression of HO1 (44). Induced HO1 reduces heme levels, which then decreases the rate of Bach1 degradation. This “feedback loop” ensures that Bach1, HO1, and Fpn levels are carefully titrated against heme level.

Bach1 appears to serve as an important transcriptional sensor of heme. Its targets include genes involved in heme degradation (for example: HO1, ferritin, and ferroportin), redox homeostasis (for example: GCLC and GCLM) as well as cell-cycle and apoptosis related (for example: CALM1, BCL2L11, and SQSTM1) (38). One notable target is the transcription factor SpiC, which is highly expressed by iron-recycling macrophages in the spleen (RPM) and is required for their development (3). SpiC is a member of the Spi subfamily of Ets transcription factor, which is also expressed in B cells (45, 46). High levels of heme in splenic red pulp were found to induce degradation of Bach1 protein in monocytes. This occurred *via* direct interaction of heme and Bach1 protein leading to proteasome-dependent degradation of Bach1, which de-repressed SpiC to promote the development of RPM from monocytes (8). This pathway is particularly important during hemolysis and conditions leading to erythrocyte damage, which requires greater number of RPM (8). Hemolysis may be associated with sterile conditions (such as sickle cell anemia) or infectious diseases (such as a traumatic wound or malaria), which are associated with distinct iron-recycling requirements. While in the sterile setting it is safe to “turn on” the RPM transcriptional program aimed at releasing the heme-associated iron back into the circulation, in infectious setting iron is usually sequestered inside the macrophages. Currently, it is unclear whether and how pathogenic stimuli with or without heme influences SpiC expression in macrophages. Finally, SpiC induction is one of the many consequences of Bach1 inhibition, and Bach1 inhibition itself is one of the many consequences of increased heme levels. The full repertoire heme’s impact on macrophage development remains to be elucidated.

## Heme-Mediated Regulation of Macrophage Activation

Heme can influence macrophage function directly or indirectly in a number of ways. Direct mechanisms include heme’s activity as a prosthetic group in hemoproteins, inhibition of Bach transcription factors, binding to nuclear receptors such as RevErb, interactions with toll-like receptor 4 (TLR4), and direct binding to DNA in the nucleus. Indirect actions are more diverse and include heme’s ability to induce ROS and generate metabolites such as iron, carbon monoxide, bilirubin, and biliverdin. The source of heme for the aforementioned activities can be cell intrinsic (all cells can produce heme) or exogenous. Here, we will restrict our discussion to the direct actions of cell-exogenous heme on macrophages.

The role of heme in inducing iron-recycling macrophages *via* Bach1 degradation is discussed above. This pathway ensures safe disposal and recycling of heme. Excess heme in circulation or tissue may also indicate damage to erythrocytes or muscle, which contains the majority of heme in the body. In this context, heme may act as an alarmin, which are endogenous factors released upon tissue damage to activate immune system (24). Heme has been shown to activate TLR4 to induce TNF production in macrophages (47). TLRs are evolutionarily conserved transmembrane receptors that recognize structurally conserved molecular motifs associated with pathogens (48). TLR4 engagement by prototypical agonist LPS activates two distinct downstream pathways: MyD88 dependent and TRIF dependent. Heme appears to selectively activate MyD88-dependent pathway and induces a different repertoire of cytokines when compared to LPS, underscoring their distinct mode of action through TLR4 (47). Notably, heme showed synergistic effects with lower doses of LPS on the production of inflammatory cytokines such as TNF and IL6 (49). This synergism required the activity of spleen tyrosine kinase and was also observed with activation of TLR2, TLR3, and TLR9 (49). This synergy may be particularly relevant to infectious diseases associated with hemolysis, such as malaria, where heme may help control infection by “boosting” TLR signaling when pathogen levels (hence TLR agonist levels) are low. Heme has also been reported to promote IL1-β processing by NLRP3 inflammasome component further supporting its role as an alarmin (50).

Macrophage activation *via* heme is also relevant in certain non-infectious diseases. Recent work has shown that heme released upon hemolysis in sickle cell disease leads to the induction of a pro-inflammatory “M1” polarization in macrophages, which was found to be dependent on TLR4 activity and ROS production (51). Importantly, exogenous administration of the heme scavenger hemopexin was found to counteract the induction of this inflammatory phenotype in macrophages of a mouse model of sickle cell disease. Heme is also a natural ligand for nuclear receptors RevErbα and RevErbβ (52–54). Heme binding to RevErbα has been shown to repress IL10 transcription in human monocytes and macrophages (55). IL10 has anti-inflammatory role and its suppression by heme RevErbα was suggested to maintain a pro-inflammatory “M1” phenotype in macrophages. Notably, genetic *cis* regulatory elements near IL10 locus promoting this action of RevErbα were present in humans but not mice (55). More global transcriptional analysis suggested that RevErbα and RevErbβ generally suppress enhancer-directed transcription in macrophages but the relevance of this in the context of heme activity in macrophage remains to be fully elucidated (56).

## Concluding Remarks

Nature has delegated macrophages the task of handling heme, which is aimed at avoiding tissue toxicity, defending against pathogens, and recycling iron to support ongoing erythropoiesis. These diverse physiological requirements involve distinct but overlapping functional modules in macrophages (Figure 1). The core functional module is the ability to “sense” and “degrade” heme. Other functional modules are context dependent. As an example, iron released upon heme degradation is exported outside the cell *via* Fpn at the steady state. During inflammation, however, Fpn is downregulated leading to an accumulation of iron inside macrophages. Indeed, macrophages excel at homeostatic functions by integrating myriad extracellular signals to formulate an appropriate physiological response.

An exciting and relatively new finding is the ability of “free” heme to act as a signaling molecule for monocyte differentiation *via* activation of the transcription factor SpiC. During inflammation or injury circulating monocytes can enter tissue to differentiate into wound-healing and anti-inflammatory macrophages or pro-inflammatory and immune-stimulatory DCs (57). Therefore, the ability of heme to influence the differentiation of a monocyte into DCs vs. macrophage has important implications on immunity and tissue homeostasis, which remains to be fully explored. In this context, it will also be important to uncover factors and pathways that control the movement of heme within distinct cellular compartments after its uptake. Several heme transporters have been described: Flvcr regulating heme export from cytoplasm, HRG-1 regulating heme transport from lysosome into cytoplasm, and Mrp5 regulating heme export from cytoplasm (58–61). Future research will likely provide new insights into pathways controlling such chaperoned movement of heme between and within cells.

Recent work has led to a gradual shift in our perception of free heme: from a mere product of erythrocyte degradation to an active signaling molecule. As future work reveals additional facets of heme activity, the number of ways in which erythrocyte-derived heme can influence macrophage development and function will grow. This may also open up new opportunities to therapeutically modulate macrophage function in various diseases.

## Author Contributions

MA and MH wrote, organized, and edited the manuscript. SD prepared the figure and helped edit the manuscript.

## Conflict of Interest Statement

The authors declare that this research was conducted in the absence of any commercial or financial relationships that could be a potential conflict of interest.
